# The Relationship of Social Support and Quality of Life with the Level of Stress in Pregnant Women Using the PATH Model

**DOI:** 10.5812/ircmj.12174

**Published:** 2013-07-05

**Authors:** Sara Shishehgar, Abolfazl Mahmoodi, Mahrokh Dolatian, Zohreh Mahmoodi, Maryam Bakhtiary, Hamid Alavi Majd

**Affiliations:** 1 Department of Midwifery, International branch of Shahid Beheshti University of Medical Sciences, Tehran, IR Iran; 2 Department of Medicine, Shahid Beheshti University of Medical Sciences, Tehran, IR Iran; 3 Department of Midwifery, Shahid Beheshti University of Medical Sciences, Tehran, IR Iran; 4 Social Determinants of Health Research Center, Alborz University of Medical Sciences, Karaj, IR Iran; 5 Department of Psychiatric, Taleghani Hospital, Shahid Beheshti University of Medical Sciences, Tehran, IR Iran; 6 Department of Biostatistics, School of Paramedical Sciences, Shahid Beheshti University of Medical Sciences, Tehran, IR Iran

**Keywords:** Pregnancy, Quality of Life, Social Support, Stress

## Abstract

**Background:**

Lack of adequate social support, stress, and generally poor quality of life during pregnancy leads to adverse pregnancy outcomes for both the mother and the baby.

**Objectives:**

This study aimed to investigate the relationship of social support and quality of life with level of stress during pregnancy.

**Materials and Methods:**

This was a descriptive-correlative study conducted on 210 pregnant women (meeting study criteria), attending Shahriar Social Services Hospital during 2012. Purposive convenient sampling was used. Study subjects completed questionnaires of obstetrics and demographics, VAUX social support, World Health Organization quality of life, and stress during pregnancy. Data were analyzed with SPSS-19 and Lisrel 8.8, utilizing statistical path analysis.

**Results:**

The final path model fitted well (CF1 = 1, RMSEA = 0.00) and showed that direct quality of life paths with β = -0.2, and indirect social support with β = -0.088 had the most effects on reduction of stress during pregnancy.

**Conclusion:**

Social support indirectly and quality of life directly affect stress during pregnancy. Thus, health officials should attempt to establish measures to further enhance social support and quality of life of pregnant women to reduce stress and its consequences during this time.

## 1. Background

Pregnancy, due to emotional, physical, and social changes, is considered an acute time in women’s lives (1). Many women are faced with stress, perhaps the greatest stress they will ever have to deal with. If other stressful events such as financial, marital, low social class issues are added to this, they could intensify its effects, leading to adverse pregnancy outcomes such as low birth weight, preterm delivery, or miscarriage (2-4). It can also cause behavioral problems, hyperactivity disorder, and attention deficit during childhood (5). This factor, through two main mechanisms of neuro-endocrine system and immune or vascular systems, and also through improper health behaviors such as smoking, drug abuse, and malnutrition, plays a role in the incidence of adverse pregnancy outcomes (6).

In studies conducted in England and Sweden, the prevalence of stress during pregnancy was reported 33-37 and 5-7 percent, respectively (7). On the other hand, connections and social networking support the person at the time of stress, making her feel valued, in control, and mentally healthy. Therefore, connections and social support during pregnancy play a very important role in the mother and the baby’s health (1). There is considerable evidence indicating the positive role of social support in people’s health and quality of life, while social isolation has been shown to result sickness (8). Many experts believe that a lack of social support, by affecting quality of life, can lead to medical and mental problems such as dyspnea, digestive problems, and depression during pregnancy and beyond (1, 9). However, support of the sexual partner can amend chronic stress effects on neonatal outcomes (10). Another factor that can influence pregnant women is social economic statues. This factor affects the overall human functioning like mental health and increases stress and depression. Also SES is a key factor in determining the quality of life so it can influence womens’ health directly or indirectly through quality of life (11). Despite previous attempts to improve psychosocial support during pregnancy and beyond for at-risk women, stress during pregnancy as a major problem still persists (12-14). Previous studies have concentrated on general pregnancy stress, and only a few have focused on pregnancy related stress and the role of social support in amending stress and improving quality of life (15).

## 2. Objectives

Given the possible effects of social support on quality of life of pregnant women, reduction of pregnancy related stress, and on improvement of pregnancy outcomes, and also, lack of availability of studies in Iran that investigate the combined role of these three factors, it was decided to conduct a study to determine the relationship of social support and quality of life with stress level in pregnant women using the PATH model.

## 3. Materials and Methods

This sectional descriptive-correlative study was conducted during 2012. Study population comprised of all pregnant women, according to convenience sampling (that met study inclusion criteria), attending any of the hospitals in west of Tehran, Iran. The required sample size, using correlation formula, was found to be 210 patients that were selected by purposeful convenient sampling.

### 3.1. Participants

#### 3.1.1. Study Inclusion Criteria

Women in their first or second pregnancy, with a single fetus, without any medical, mental, or disabled spouse or child, and no major life event in their last month of pregnancy, non-smoker or drug user and those who had performed necessary pregnancy care.

### 3.2. Measurement Questionnaires

Data were collected through distinct pregnancy stress, summarized WHO quality of life, modified social support VAUX (SS-A), and demographic questionnaires.

#### 3.2.1. Demographic and Obstetrics Questionnaire

This included woman’s age, spouse’s age, number of children in terms of gender, pregnant woman’s and spouse’s opinion of pregnancy status, current marital status, pregnant woman’s and spouse’s occupation, obstetric history including; gestational age, number of pregnancies, number of births, parity, living children, first day of last menstrual period, time interval to last pregnancy or miscarriage, history of preterm birth, intrauterine fetal death, consumption of alcohol and exposure to tobacco smoke. To assess the socio-economic status, a questionnaire was designed including the education level of the pregnant mother, the education level of the spouse, their place of residence and number of people per household, cost per square meter of their house, facilities and leisure (having a private car and computer). In this questionnaire, the correlation of these parameters with total score was found to be 0.87. Using factor analysis and summary index, the total standardized score for all subjects was calculated, and using the Kappa test, its compliance with normal summary index was investigated. Therefore, the potential maximum score in the summarized index was 46 marks (16).

#### 3.2.2. The WHO QOL–BREF Questionnaire

In this study, the validated Iranian version of the WHO QOL questionnaire was used (WHO QOL-BREF). It is a short version of the 100-scale instrument, comprising 26 items, and reflects the multi-dimensional nature of QOL; it also emphasizes subjective experiences rather than objective life conditions and it focuses upon the respondent’s perceived QOL (17). The WHO QOL-BREF was developed for a wide range of cultural and clinical settings (18-20). It contains four domains, namely physical health, psychological status, social relationships and environmental conditions (21). Each question scores 1-5 in Likert style, and the total score was changed in to percentile and classified into: poor (0-33.3%), average (33.4-66.3%), and desirable (66.4-100%). An Iranian reliability study showed that Cronbach's alphas for the four domains of the WHO QOL-BREF were satisfactory (physical health = 0.81, psychological status = 0.78, social relationships = 0.82, and environmental conditions = 0.80) (3) .The domain score was converted to a transformed score (ranging from 4 to 20) to enable comparison between domains. A higher score denotes a higher QOL. The domain scores were computed on the basis of WHO profiles (21).

#### 3.2.3. Social Support Questionnaire

The theoretical basis of VAUX (SS-A) social support scale was found on the basis of Cobb’s definition (1997) of social support. This scale contains 23 items in three domains of family, friends, and associates, with 8, 7, and 8 questions in each domain. This questionnaire has been designed with 4-point Likert style of strongly disagree, disagree, agree, and strongly agree, and scores are calculated for each of the social support domains of family, friends, associates, and also the overall social support score, which is the sum of the scores of these domains. Validity and reliability of this questionnaire has been determined by previous studies. People with ≥ 18 have greater social support than those with scores ≤ 18. Validity of the social support questionnaire through content validity, and it reliability through Cronbach’s alpha 0.7-0.9 were determined by Ebrahimi-Ghavam in 1982.

#### 3.2.4. Specific Stress Specified by the Pregnancy Questionnaire

**Figure 1.**
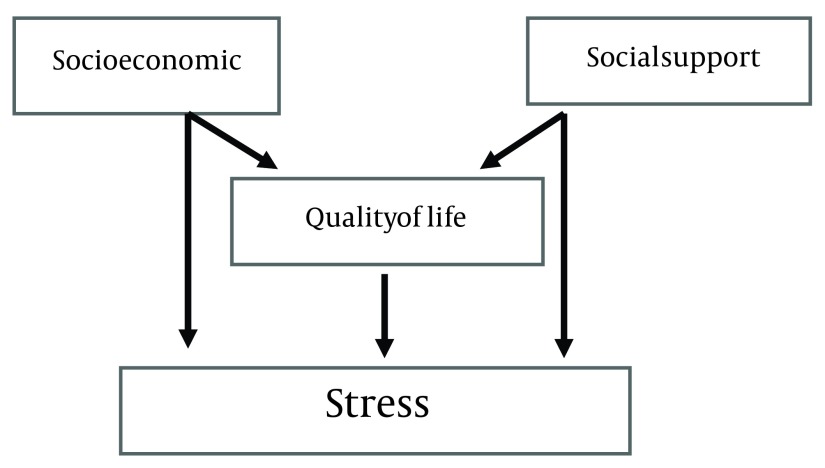
Theoretical Path Model for Effects of Quality of Life, Social Support and Stress During Pregnancy

A questionnaire consisting of 51 questions in 6 domains, of others think that individual, financial, religious, environmental, personal, family and health. This questionnaire has been designed with 5-point Likert style of zero (minimum) to 204 (maximum). In all areas grading is done as follows 0 = 1, low = 1, medium = 2, high = 3 and very high = 4. After data collection and conversion, numbers are classified into three grades: mild stress = 0-33.3, average stress = 33.4-66.3, severe stress = 66.4-100. Validity and reliability of the Specific stress in pregnancy questionnaire has been determined by salari et al. with test-retest and Cronbachs alpha coefficient = 0.75 ( 22 ). In this study, the fitness of a conceptual path analysis model for assessing social support parameters concurrent relationship with quality of life and level of stress in pregnant women was investigated (Figure 1). 

Path analysis method is a generalization of normal regression, which in addition to expressing direct effect, shows indirect effect as well as effects of each parameter on dependent parameters, and using these results, a rational explanation of the observed relationships and correlations can be provided. The SPSS19 (x 2 and ANOVA) and Lisrel 8.8 software were used for analysis of data with application of path analysis. To comply with ethical considerations, permissions for conducting this study were obtained from the Shahid Beheshti University Chancellor and also, the Director of the selected hospital. Also, before commencement, mothers were informed about the study aims and consented to take part. They were assured of the confidentiality of their information, and were told that they could withdraw from the study any time they wished. We also considered the patient’s privacy.

## 4. Results

In this study, 210 pregnant women with mean age 29 ± 4.8 years took equal parts in three trimesters of pregnancy. The majority (90.5%) were city dwellers, educated at high school diploma level (51.4%), housewives (89%), Azari (40.5%), and had wanted/planned pregnancies (78.6%) (Table 1). 

**Table 1.** Characteristics of Pregnant Women (n = 210) in a Hospital in the West of Tehran

Variables	No, (%)	Mean ± SD
**Age, yr**		29 ± 4.8
< 25	87 (41.4)	
25-30	73 (34.8)	
30-35	40 (19)	
> 35	10 (4.8)	
**Education, yr**		
< 12 years	155 (73.8)	
12-16 years	55 (26.2)	
**Job**		
Housekeeper	187 (89)	
Employed	23 (11)	
**Income**		
< 50000000 R	13 (65.5)	
> 50000000 R	10 (43.5)	
**Parity**		
1	107 (51)	
2	103 (49)	

Mean scores were as follows; social support during pregnancy 17.2 ± 3, quality of life 64.4 ± 11.3, and pregnancy stress 43.6 ± 20.1, indicating acceptable social support, average quality of life, and average pregnancy stress. There was a significant correlation between social support and quality of life (P < 0.001). According to our results, there was a significant correlation between socio economic statues and quality of life and stress during pregnancy (P < 0.001) but it didn’t have any significant relationship with social support (Table 2). 

**Table 2.** The Relation Between Quality of Life and Social Support

Social Support	Mean ^a^ ± SD	ANOVA
**Quality of Life**		
Poor	7 ± 0	P < 0.001
Medium	16.3 ± 3	
Good	18.5 ± 2.5	

^a^ Mean is 0-23

To perform path analysis, correlations between parameters were found using Bivariate Analysis. Stress was inversely and significantly correlated with social support and quality of life. Also, social support showed direct and significant correlation with quality of life (Table 3). 

**Table 3.** Correlations Between ****Quality of Life, Social Support, Socio Economic Status and Stress During Pregnancy

	Quality of Life	Social Support	Socio Economic Status	Stress During Pregnancy
**Quality of Life**	1	0.432	0.019	-0.230
**Social Support**		1	0.093^a^	-0.139
**Socio Economic Status**			1	0.046
**Stress During Pregnancy**				1

^a^ No Significant

The indices GFI, CFI, and RMSEA were used to investigate the model fitness. The results indicated desirability, high fitness, and rationality of the parameter relationships based on the conceptual model. Accordingly, the fitted model had no significant difference with the conceptual model (Table 4). 

**Table 4.** Goodness of Fit Indices for the Model

	X^2^	df	P	GFI	CFI	RMSEA
**Model index, n = 210**	0.44	1	0.51	1	1	0.000

**Figure 2.**
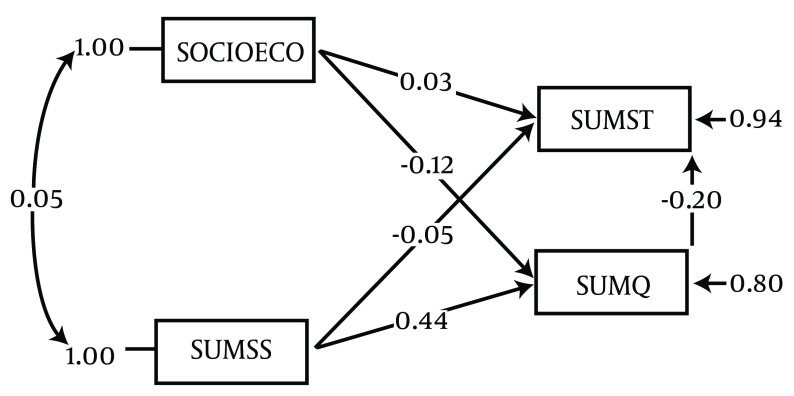
Full Empirical Model (Empirical Path Model for Empirical Path Model for Relationship of Social Support With Quality Of Life and Level of Stress In Pregnant Women)

According to the path diagram, among direct paths, only path of quality of life (β = -0.2) had a significant effect on pregnancy stress, and for indirect paths, social support (β = -0.088) had the most effect on stress. The results of the model indicated that favorable social support and quality of life have a stress reducing effect during pregnancy, and unfavorable socio-economic status increases stress (Table 5) (Figure 2). 

**Table 5.** Path Coefficients for Quality of Life, Social Support and Stress During Pregnancy

Predictor Variables	Effects			Model Coefficients	t value
	Direct	Indirect	Total		
**Socio-Economic**	0.03 ^a^	0.024	0.054	0.26	1.89
**Social Support**	-0.05 ^a^	-0.088	-0.138	1.69	7.3
**Quality of Life**	- 0.20		-0.20	0.73	2.72

^a^ No Significant

## 5. Discussion

In this study, attempts were made to create a strong relationship between theoretical and applied issues of research using path analysis. Accordingly, the results of the model indicated that quality of life had the most direct and reducing effect on stress during pregnancy. This shows that the better the quality of life, the lower the level of pregnancy stress. Quality of life during pregnancy is measurable (21), and given its positive effect in preventing adverse pregnancy outcomes by various ways such as reducing stress, its measurement is necessary for planning care for mothers and babies, and understanding the necessity of this is highly important for policy makers and healthcare organizations. A good quality of life roots in religious beliefs and learning among Muslims. One of these factors is social support.

In the present study, social support, through indirect effect on quality of life, had the most effect (next to quality of life) on pregnancy stress. This finding was in line with Elsenbruch results (2007) reporting that a lack of social support adversely affected mother’s mental state during pregnancy (1). Gabbe et al. (2012) believe social support can change quality of life of pregnant women, and if unfavorable, it leads to heartburn, nausea, vomiting, cramp in the legs, and shortness of breath (9). In the opinion of Brummet et al. (2005), social support plays an important role in health, and ultimately in the quality of life of people of the community (8). Social support as an emotional coping mechanism has the potential power to affect quality of life. Social support can be present in the forms of emotional and mental support and information while it can be tangible and sociable (23). Whilst, life’s social dimension has a significant effect on health in general, and on quality of life in particular (24). Social support during pregnancy is considered a necessary factor in health and well-being of mothers. Women with ample social support, despite high levels of stress, have fewer complications during pregnancy, and stress in pregnant women increases with decreasing social support. Care for women with risk factors of stress, and also counseling and referral for diagnosis and advanced treatments, can improve their quality of life (25). In the present study, socio-economic status had an increasing effect on mother’s stress during pregnancy. Hawamdeh et al. also believe that material deprivation and economic inequality through psycho-social factors, lifestyle behaviors, and physiopathological changes have very important effects on the incidence of chronic diseases and mental health of people (26). Families at lower socioeconomic levels are faced with problems such as malnutrition, inadequate pregnancy care, addiction, smoking, alcohol, frequent pregnancies, stress, etc, which can lead to adverse pregnancy outcomes (27, 28).

Positive points of this study were the consideration of three trimesters of pregnancy and it could be omitted the forgotten problem. In this study, we considered a hospital in the west of Tehran that may be different to other parts so we suggest that the same study should be done for other parts of Tehran and comparisons be made. Generally, this study showed that social support indirectly through directly affecting quality of life influences stress during pregnancy. Thus, health officials should attempt to establish measures to further enhance social support and quality of life of pregnant women, to reduce stress and its consequences during pregnancy.
